# Structural insights into multifunctionality of human FACT complex subunit hSSRP1

**DOI:** 10.1016/j.jbc.2021.101360

**Published:** 2021-10-28

**Authors:** Xuehui Li, Huiyan Li, Qian Jing, Mengxue Wang, Tingting Hu, Li Li, Qiuping Zhang, Mengxin Liu, Yu Vincent Fu, Junhong Han, Dan Su

**Affiliations:** 1 State Key Laboratory of Biotherapy and Cancer Center, West China Hospital, Sichuan University and Collaborative Innovation Center of Biotherapy, Chengdu, Sichuan, China; 2 Research Laboratory of Tumor Epigenetics and Genomics, Department of General Surgery, Frontiers Science Center for Disease-related Molecular Network, State Key Laboratory of Biotherapy, and National Clinical Research Center for Geriatrics, West China Hospital, Sichuan University, Chengdu, China; 3 State Key Laboratory of Microbial Resources, Institute of Microbiology, Chinese Academy of Sciences, Beijing, China; 4 College of Life Sciences, Neijiang Normal University, Neijiang, Sichuan, China; 5 Infectious Disease Drug Discovery Institute, Tianjin International Joint Academy of Biotechnology and Medicine, Tianjin, China

**Keywords:** hSSRP1, histone chaperone, FACT complex, HMG domain, PH domain, CK2, casein kinase II, CTD, C-terminal disordered region, FACT, facilitates chromatin transcription, HBD, H2A–H2B binding region, HMG, high-mobility group, hSSRP1, human structure-specific recognition protein 1, ITC, isothermal titration calorimetry, MBD, minimal binding domain, MD, middle domain, NLS, nuclear location signal, PH, pleckstrin homology, Pob3, polymerase one binding protein 3, RFP, redfluorescent protein, SEC, size-exclusion chromatography

## Abstract

Human structure-specific recognition protein 1 (hSSRP1) is an essential component of the facilitates chromatin transcription complex, which participates in nucleosome disassembly and reassembly during gene transcription and DNA replication and repair. Many functions, including nuclear localization, histone chaperone activity, DNA binding, and interaction with cellular proteins, are attributed to hSSRP1, which contains multiple well-defined domains, including four pleckstrin homology (PH) domains and a high-mobility group domain with two flanking disordered regions. However, little is known about the mechanisms by which these domains cooperate to carry out hSSRP1’s functions. Here, we report the biochemical characterization and structure of each functional domain of hSSRP1, including the N-terminal PH1, PH2, PH3/4 tandem PH, and DNA-binding high-mobility group domains. Furthermore, two casein kinase II binding sites in hSSRP1 were identified in the PH3/4 domain and in a disordered region (Gly^617^–Glu^709^) located in the C-terminus of hSSRP1. In addition, a histone H2A–H2B binding motif and a nuclear localization signal (Lys^677^‒Asp^687^) of hSSRP1 are reported for the first time. Taken together, these studies provide novel insights into the structural basis for hSSRP1 functionality.

Eukaryotic genomic DNA is organized into densely packed chromatin, a higher-order architecture dominated by arrays of basic repeating units, termed nucleosomes (1, 2). The structure of the nucleosome consists of 146 bp of DNA wrapped around an octamer of histone proteins, which comprised a heterotetramer of histones H3-H4 and two heterodimers of histones H2A-H2B (3). Depending on the organism and cell type, up to 75 to 90% of eukaryotic DNA is wrapped around consecutive histone octamers, which represent barriers to DNA replication, repair, and transcription machinery (4). Therefore, nucleosomes play a central role in the compaction of genomic DNA and the control of DNA accessibility for transcription and replication. In the regulation of nucleosome dynamics, nucleosome disassembly and reassembly are two critical processes that occur periodically (5). To regulate these molecular events, cells have evolved many nucleosome-associated factors and regulatory mechanisms that coordinate nucleosome disassembly and assembly, including ATP-dependent chromatin remodelers and ATP-independent histone chaperones (6, 7, 8). The FACT (facilitates chromatin transactions) complex, a heterodimer composed of the subunits (structure-specific recognition protein-1) SSRP1 and Spt16, is an abundant and conserved histone chaperone complex found in all eukaryotes (9, 10, 11). It is also an essential ATP-independent histone chaperone complex that allows eukaryotic RNA polymerase II to transcribe chromatinized DNA by destabilizing nucleosomes before the arrival of the polymerase (12) and restabilizing them after transcription (13, 14, 15). Previous studies have found that FACT binds specifically to mono-nucleosomes *via* interactions with both histone H2A–H2B dimers, (H3–H4)_2_ tetramers, and DNA, playing critical roles in reversible nucleosome reorganization (16, 17, 18, 19).

hSSRP1, an 87 kDa subunit of the FACT complex, is highly conserved across all eukaryotes, except for its high-mobility group (HMG) domain, which is present in hSSRP1 (human SSRP1) but absent in the yeast homolog Pob3 (polymerase one binding protein 3) (20, 21, 22). The function of the missing HMG domain in yeast is provided by the small HMG-box protein, Nhp6. hSSRP1 was initially characterized as an HMG-box protein binding to cisplatin-modified DNA with a classic DNA-binding domain at its C terminus, which enables it to bind DNA as it interacts with the nucleosome (23). The DNA-binding activity of hSSRP1 is negatively regulated by casein kinase II (CK2)-mediated phosphorylation of hSSRP1 at its HMG-containing C-terminal region, leading to a reduction in DNA interactions. CK2 may inhibit hSSRP1 function in transcriptional elongation and replication, thus preventing its association with chromatin (24, 25). A kinase complex containing CK2 and the SSRP1–Spt16 complex has been previously found to alter the specificity of CK2 in the complex such that it selectively phosphorylates p53 over other substrates (26). Previous studies have shown that, in addition to the FACT function, hSSRP1 exhibits Spt16-independent functions in the regulation of gene transcription (27, 28). A special role of hSSRP1 was discovered in the regulation of the activity of transcription factors, including p63 and serum response factor (29, 30). hSSRP1 also facilitates microtubule growth in mitotic cells (31) and promotes the activation of the Wnt/β-catenin-mediated signaling pathway during cellular differentiation (32). Furthermore, hSSRP1 is involved in latency-associated nuclear antigen-dependent DNA replication, which interacts with telomeric repeat binding factor 2 to form complexes with latency-associated nuclear antigen (33). hSSRP1 also associates with the export adapter UAP56-interacting factor, participating in the export of cellular mRNAs (34).

As a multifunctional protein, although hSSRP1 has highly conserved core domains, it differs slightly among single-cell eukaryotes, plants, and metazoans because of the variable inclusion of domains (35). Collectively, hSSRP1 consists of an N-terminal domain, middle domain (MD), internal intrinsically disordered domain, HMG box, and a C-terminal intrinsically disordered domain (36). The hSSRP1 N-terminal domain is responsible for interaction with SPT16, which contains two PH motifs (PH1 and PH2) (37). MD is a double pH domain (PH3/4) that is likely to interact with histone H3–H4 because of its similarity with the known H3-H4 chaperone Rtt106 (38) and being involved in DNA binding (39). The HMG domain is a sequence-independent DNA-binding motif that belongs to the HMGB family and has a high affinity for kinked or bent DNA (23, 40). The C-terminal intrinsically disordered domain has been reported to bind to left-handed Z-DNA (41). After a decade of effort, a large number of functional segments and isolated structural domains have been described for hSSRP1 and its homologs. These homologs include structures of the hSSRP1 middle domain (PDB code 4IFS) (39), HMG domain of SSRP1 in fruit fly (PDB code 1WXL) (42), Spt16 dimerization domain of Pob3 in yeast (PDB code 3F5R), and Pob3 middle domain (PDB code 2GCL) (43). In particular, very recently, two cryo-EM structures of human FACT complexed with partially assembled “sub-nucleosomes” were observed, revealing the structural basis of FACT-mediated nucleosome disassembly (44). Despite these observations, the mechanism by which the multiple domains cooperate to carry out the functions of hSSRP1 has yet to be fully elucidated. In this study, we report the biochemical characterization and structure of each functional domain from hSSRP1, including the N-terminal PH1, PH2, PH3/4 tandem PH, and DNA-binding HMG domains. Furthermore, we also identified several functional regions on hSSRP1, including the H2A–H2B binding region, two CK2 binding sites, and the nuclear location signal (NLS)-mediated nuclear localization of hSSRP1. Based on these results, an overview of the topology of the full-length hSSRP1 has been proposed.

## Results

### Structure of tandem PH domain in hSSRP1

**Figure 1:**
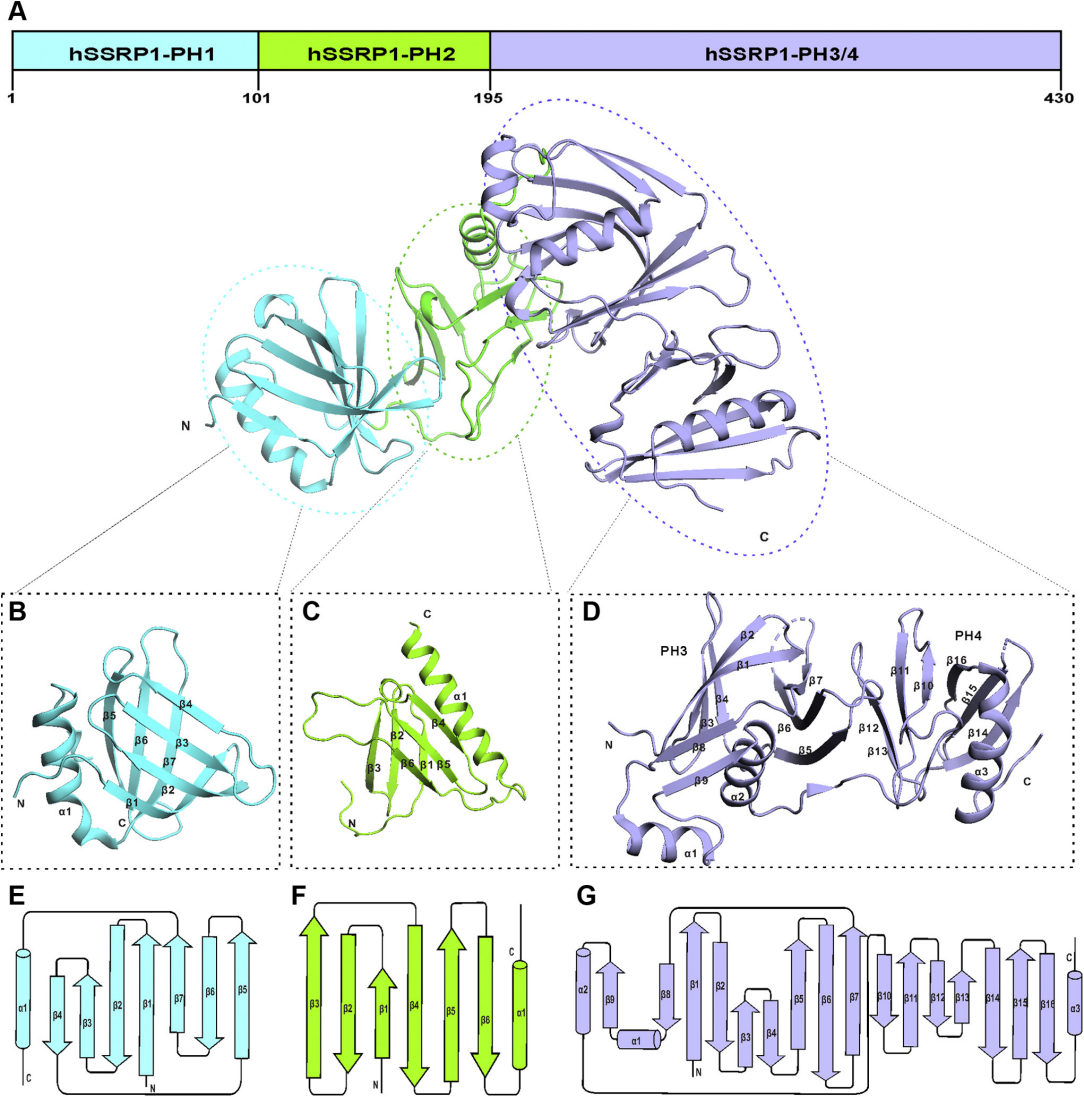
**Structural overview of N-terminal tandem PH domain in hSSRP1.***A*, ribbon representation of the model of N-terminal hSSRP1 contains four PH domains including hSSRP1-PH1, -PH2, and -PH3/4. The structure-based domains are shown as *cartoons* in *aqua*, *light green*, and *lavender blue*. *B*, the structure of hSSRP1-PH1 (Met^1^-Asp^100^). An orthogonal view of the PH1 domain is shown with labeled-secondary structural elements. *C*, the model of hSSRP1-PH2 (Leu^101^-Thr^195^) is colored in *light green*. *D*, the structure of hSSRP1-PH3/4 (Gly^196^-Asn^430^) consists of two PH domains connecting with a loop. *E*–*G*, a topology diagram of the protein fold of hSSRP1-PH1, hSSRP1-PH2, and hSSRP1-PH3/4. hSSRP1, human structure-specific recognition protein; PH, pleckstrin homology.

The sequence of WT hSSRP1 (Met^1^–Glu^709^) was submitted to a template-based algorithm of ThreaDom (45) and FUpred server (46) for predicting domains and intrinsically disordered regions. The outputs indicated that hSSRP1 contained four PH domains in the N-terminus (Met^1^– Arg^428^) followed by the HMG domain within a long disordered region (Ala^431^–Glu^709^). In addition, we combined the hydrophilicity of amino acids and the secondary structure to fine-tune the boundary of four PH domains. For the convenience of description, we named the three subdomains hSSRP1-PH1 (Met^1^–Asp^100^), -PH2 (Leu^101^–Thr^195^), and -PH3/4 (Gly^196^–Asn^430^). We first attempted to determine the structure of the N-terminal hSSRP1 covering the PH1-4 domains using X-ray crystallography. Despite numerous trials, we were unable to get the crystals of PH1-4. However, we obtained high-quality crystals of PH1 and PH3/4. After solving the PH1 and PH3/4 subdomain structures, we built a model of the tandem PH domain hSSRP1-PH1-4 (Met^1^–Asp^430^) by using Robetta (47). As shown in Figure 1*A*, the split PH1, PH2, PH3, and PH4 adopt a canonical PH domain fold.

The structure of hSSRP1-PH1 (Met^1^–Asp^100^) was determined to a resolution of 1.8 Å, with two molecules in the asymmetric unit. The final R-factor of the structural model was 0.17, whereas R_free_ was 0.21. In total, 99.0% of the residues fell in the favored regions of the Ramachandran plot, whereas 1.0% fell in the disallowed regions. The data collection and refinement statistics are presented in Table 1. The structure of hSSRP1-PH1 comprised a seven-stranded antiparallel β-barrel capped by a C-terminal helix (Fig. 1, *B* and *E*). Similar structures were collected from *Saccharomyces cerevisiae* Pob3-N (scPob3-N, PDB ID: 3F5R) and *Chaetomium thermophilum* Pob3-N (CtPob3-N, PDB ID: 4KHB) (Fig. S1*A*). Superposition of the hSSRP1-PH1 domain with Pob3-N proteins revealed RMSDs of 1.44 and 1.86 Å for all C^a^ atoms between hSSRP1-PH1 and scPob3-N and hSSRP1-PH1 and CtPob3-N, respectively (Table S1). Size-exclusion chromatography (SEC) and electrophoretic mobility shift assays (EMSAs) were used to determine the properties of the hSSRP1-PH1 protein. Molecular weight calibration experiments revealed a molecular weight of 12 kDa, close to the theoretical molecular weight of the hSSRP1-PH1 monomer (Fig. S1*B*). Meanwhile, no evidence was found for the interaction of hSSRP1-PH1 with histones, including the H2A–H2B dimer, H3–H4 tetramer, and H1 (Fig. S1, *C*–*F*). The EMSA assays showed that hSSRP1-PH1 is unable to bind dsDNA (Fig. S1*G*).

**Table 1:** X-ray crystallography data collection and refinement statistics

Data set	hSSRP1-PH1 PDB code 6L1R	hSSRP1-PH3/4 PDB code 6L1E	hSSRP1-HMG PDB code 6L34
Data collection			
Wavelength (Å)	1.5406	1.0000	1.5406
Space group	*P*43	P212121	P62
Unit-cell parameters (Å, °)	a = b = 37.82 and c = 68.09 α = β = γ = 90.00	a = 39.00, b = 66.21, and c = 84.50 α = β = γ = 90.0	a = b = 64.99 and c = 55.07 α = β = 90.0 and γ = 120.0
Resolution (Å)	50.00–1.80 (1.88–1.80)	50.00–2.10 (2.18–2.10)	55.76–2.00 (2.07–2.00)
Average *I/σ(I)*	18.16 (6.89)	26.94 (3.83)	37.39 (7.73)
No. of observed reflections	29,296 (2152)	97,951 (7058)	65,072 (6292)
No. of unique reflections	8686 (873)	13,418 (1217)	8897 (883)
Completeness (%)	95.7 (82.7)	99.1 (92.1)	99.1 (100)
Multiplicity	3.4 (2.5)	7.3 (5.8)	7.4 (7.1)
Matthews coefficient (Å^3^Da^−1^)	2.08	2.02	4.08
Solvent content (%)	41.02	39.16	69.85
Molecules per asymmetric unit	1	1	1
Refinement			
R_work_/R_free_	0.17/0.21	0.19/0.22	0.17/0.20
Ramachandran favored (%)	98.99	99.08	100
Ramachandran allowed (%)	1.01	0.92	0
Ramachandran outliers (%)	0	0	0
No. of atoms			
Protein	101	232	68
Water	197	85	108
RMS deviations			
Bond lengths (Å)	0.008	0.007	0.007
Bond angles (°)	1.058	0.865	0.729

The second PH domain, hSSRP1-PH2 (residues Leu^101^–Thr^195^) was modeled using Robetta (47) because of its high dynamics and instability in solution, preventing crystallization. This PH domain is necessary for interaction with the dimerizing domain of hSpt16. It is highly conserved in the hSSRP1 homolog Pob3, which is responsible for heterodimerization with SPT16 in yeast (37). The SEC result showed that the hSSRP1-PH2 had a molecular weight of 46.5 kDa, close to the theoretical molecular weight of the hSSRP1-PH2 tetramer (Fig. S2*A*). A homology model was built for the hSSRP1-PH2 protein from the yeast Pob3 (PDB code: 4KHB) using the Robetta server (Fig. 1, *C* and *F*). The stereochemical quality of the models selected for further analysis was good, with 92.5% of the modeled residues in the most favored regions of the Ramachandran plot (Fig. S2*B*). We further assessed the model using the QMEAN-Z (−0.53) (48) and ProSA (−3.7) server (49), which indicated relatively good model quality (Fig. S2, *C* and *D*). The hSSRP1-PH2 model contained a classic PH fold with six β-sheets (β1–6) and a short α-helix (α1). The EMSA assays showed that hSSRP1-PH2 is unable to bind dsDNA or ssDNA (Fig. S2*E*). The SEC results suggested that there is no interaction between hSSRP1-PH2 with other PH domains, including hSSRP1-PH1 and hSSRP1-PH2, hSSRP1-PH2, and hSSRP1-PH3/4 (Fig. S2*F*).

The last two PH domains hSSRP1-PH3/4 (MD domain, residues Gly^196^–Asn^430^) are expressed as a single recombinant protein, the structure of which was determined at 2.1 Å resolution by using single-wavelength anomalous diffraction. The crystallographic statistics are summarized in Table 1. The structure contains two PH domains connected by a disordered loop. In comparison with the PH4 domain, which resembled the classic PH domain, the PH3 domain has two extra antiparallel strands (β8 and β9) linked by a helix (α1), which is stretched out from the PH fold by connecting β7 and helix α2 (Fig. 1, *D* and *G*). This structure of hSSRP1-PH3/4 is similar to the human SSRP1-M (PDB code 4IFS), which was reported by Zhang *et al.* (39). Comparing these two structures with the yeast homolog Pob3 (PDB ID 4PQ0) and the related protein Rtt106 (PDB ID 3GYP), the topology of hSSRP1-PH3/4 is highly conserved with Pob3 (Fig. S3*A* and Table S1). Molecular chromatography calibration data showed that hSSRP1-PH3/4 was monomeric in solution (Fig. S3*B*) and was unable to bind to histones H2A–H2B or H3–H4 (Fig. S3, *C* and *D*). A previous study reported that hSSRP1-PH3/4 can bind short dsDNA nonspecifically *via* one positively charged patch on the surface of the structure (39). Therefore, it is of interest to determine whether the length of dsDNA influences the interaction with hSSRP1-PH3/4. We prepared different length dsDNA (15 and 30 bp) and mixed them with hSSRP1-PH3/4, respectively, at different concentrations to detect protein–DNA interactions by using EMSA. As shown in Fig. S3, *E* and *F*, hSSRP1-PH3/4 prefers to interact with dsDNA of 30 bp in length, suggesting that the length of dsDNA may dictate the DNA‒protein binding mode. Furthermore, the interaction between biotin-labeled lambda-phase DNA (48,502 bp, linear and double-stranded DNA) and hSSRP1-PH3/4 was not observed by fluorescence microscopy (Fig. S3, *E*–*G*). These results suggest that the DNA-binding ability of hSSRP1-PH3/4 is length dependent.

### hSSRP1 histone H2A–H2B binding motif

**Figure 2:**
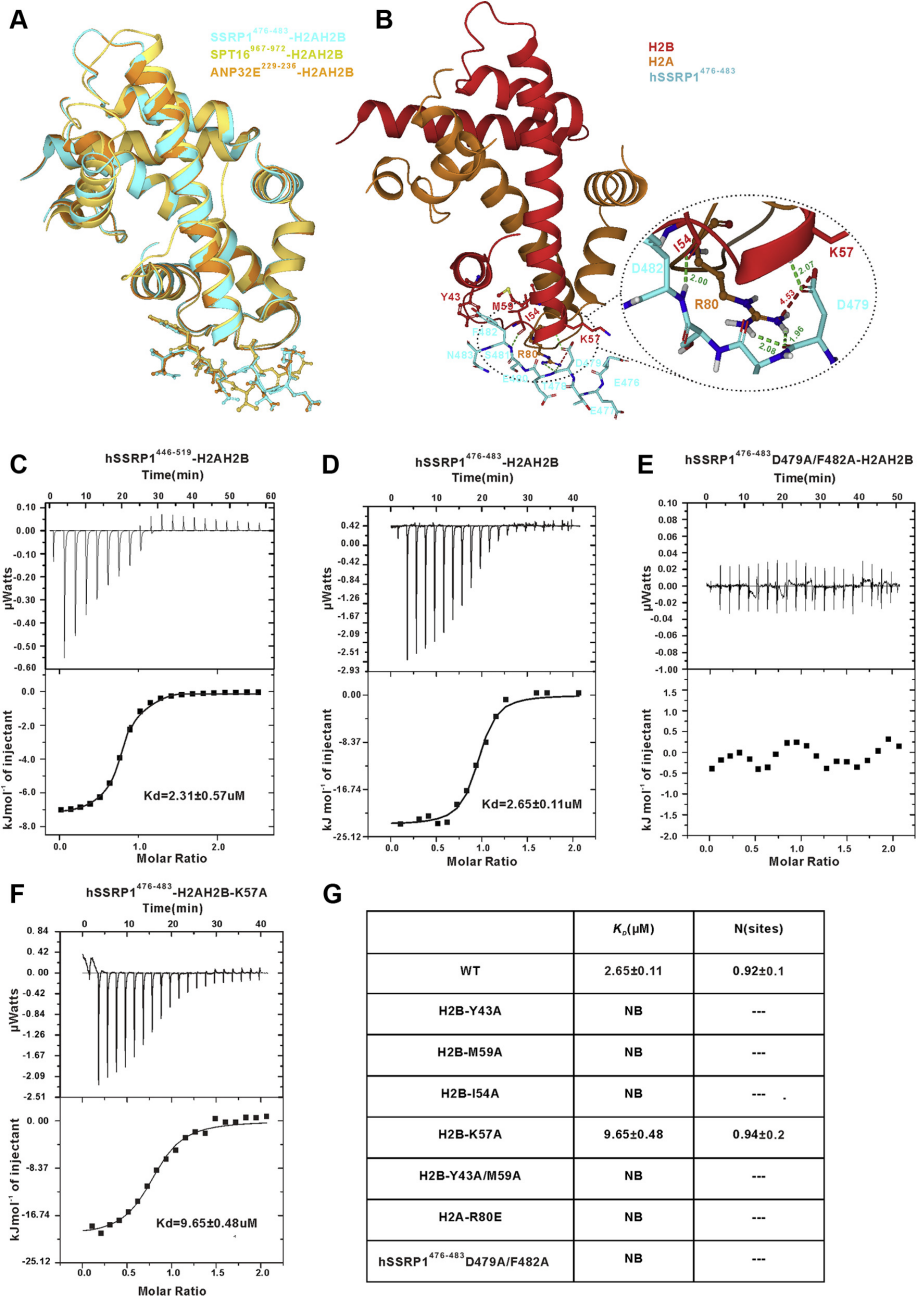
**The structural model of hSSRP1**^**476–483**^**‒H2A–H2B complex and ITC profiles of the interaction between hSSRP1**^**476–483**^**peptide and H2A-H2B variants.***A*, the structural superposition of hSSRP1^476–483^‒H2A–H2B complex, ANP32E^229–236^‒H2A–H2B complex (PDB: 4CAY), and SPT16^967–972^‒H2A–H2B complex (PDB: 4WNN). *B*, the structural model of hSSRP1^476–483^‒H2A–H2B complex. H2A are colored *orange*. H2B are colored *red*. hSSRP1 are colored *aquamarine*. The *Green-colored dotted line* indicates hydrogen bond, the *red-colored dotted line* indicates Salt bridge. *C*–*G*, ITC data for the titrations of various hSSRP1^476–483^ into H2A–H2B variants. *C*, hSSRP1-HBD titrated into H2A–H2B. *D*, hSSRP1^476–483^ titrated into H2A–H2B. *E*, the mutant hSSRP1^476–483^-D479A/F482A titrated into H2A–H2B. *F*, hSSRP1^476–483^ titrated into the mutant H2A–H2B-K57A. *G*, the binding affinities (mean *K*_*D*_ and SDs) and stoichiometry (N, sites) determined by ITC. The affinities are for full-length H2A-H2B or a variant that lacks the first 26 residues of H2B. NB, no binding. Raw data (*upper panel*) and integrated curves data (*lower panel*) are shown. HBD, H2A–H2B binding region; hSSRP1, human structure-specific recognition protein; ITC, isothermal titration calorimetry.

The previous studies have found that Pob3, the homolog of hSSRP1 in yeast, can bind with H2A–H2B at a 1:1 stoichiometry both *in vitro* and *in vivo* (50, 51). Based on the sequence alignment result, we predicted that hSSRP1 has an H2A–H2B binding region that lies between residues E^446^ and Q^519^ (Fig. S7). To clarify the H2A–H2B binding region in hSSRP1 (hSSRP1-HBD), analytical gel filtration and isothermal titration calorimetry (ITC) were applied. To this end, the full-length H2A–H2B or truncated H2A–H2B^27–126^, which lacks eight basic residues at the N-terminus of histone H2B were mixed with hSSRP1^446–519^ respectively and injected on a Superdex 200 10/300GL column. The mixed proteins were found to elute at an earlier elution volume compared with the volume of hSSRP1^446–519^, indicating that hSSRP1^446–519^ directly interacts with the H2A–H2B dimer independently of the N-terminal tail of histone H2B (Fig. S4, *A* and *B*). Furthermore, the record ITC binding curve of hSSRP1^446–519^ with H2A–H2B was exothermic, with a *K*_*D*_ value of 2.31 ± 0.57 μM with 1:1 stoichiometry (Fig. 2*C*). Based on these results, we propose that hSSRP1 contains a conserved H2A–H2B binding motif across species, which may recapitulate the full H2A–H2B binding activity of hSSRP1.

We predicted that the H2A–H2B minimal binding motif (MBD) of hSSRP1 could be narrowed down from the region of Glu^446^–Gln^519^. Several synthetic peptides covering this region were used to measure the binding ability of H2A–H2B. A peptide (^476^EETDESFN^483^), which was highly conserved in the homologs of hSSRP1 retained nearly the full binding affinity, with a *K*_*D*_ value of 2.65 ± 0.11 μM with 1:1 stoichiometry by ITC analysis (Fig. 2*D*). Therefore, this peptide represents the primary binding site for H2A–H2B in hSSRP1. To further understand the mechanism of hSSRP1 binding, we obtained the structure model of the hSSRP1^476–483^ complex with H2A–H2B^27–126^ using the docking program of AutoDock (Fig. 2*B*). The best hSSRP1^476–483^-H2A–H2B model (biding energy value: −69.45 Kcal/mol) superimposing with the structures of Spt16^967–972^-H2A–H2B complex (PDB: 4WNN) and ANP32E^229–236^-H2A–H2B complex (PDB: 4CAY) suggests H2A–H2B interaction associated with the residue hSSRP1-Asp^479^, which forms a salt bridge and two hydrogen bonds with H2A-Arg^80^ and one hydrogen bond with H2B-Lys^57^. Meanwhile, another highly conserved residue hSSRP1-Phe^482^ nestles into a hydrophobic pocket formed by the H2B residues Tyr^43^ and Met^59^ and forms a hydrogen bond with Ile^54^, playing a pivotal role in hSSRP1^476–483^-H2A–H2B interaction (Fig. 2, *A* and *B*). Therefore, we evaluated whether residues Asp^479^ and Phe^482^ in hSSRP1-MBD are critical for binding H2A-H2B. First, the peptide (^476^EETAESAN^483^) of hSSRP1 with two residue mutations (D479A and F482A) was titrated in H2A-H2B^27–126^, which was detected with no interaction by using ITC (Fig. 2*E*). We then prepared H2A–H2B mutants H2B-Y43A, H2B-M59A, H2B-K57A, H2B-I54A, H2A-R80E, and H2B-Y43A/M59A to detect the binding ability with hSSRP1-MBD following the same conditions. The results showed that the mutants H2B-Y43A, H2B-M59A, H2B-Y43A/M59A, H2B-I54A, and H2A-R80E lost their binding ability with hSSRP1^476–483^. The mutant H2B-K57A caused a marked decrease in the interaction with a *K*_*D*_ value of 9.65 ± 0.48 μM (Figs. 2, *F* and *G* and S4, *C*–*G*). Together, these data indicated that the H2A–H2B binding site was distributed over the MBDs in hSSRP1-HBD and that residues hSSRP1-Asp^479^, hSSRP1-Phe^482^, H2B-Tyr^43^, H2B-Met^59^, H2B-Ile^54^, H2B-Lys^57^, and H2A-Arg^80^ made a major contribution to the hSSRP1-H2A–H2B interaction.

### HMG related DNA-binding of hSSRP1

**Figure 3:**
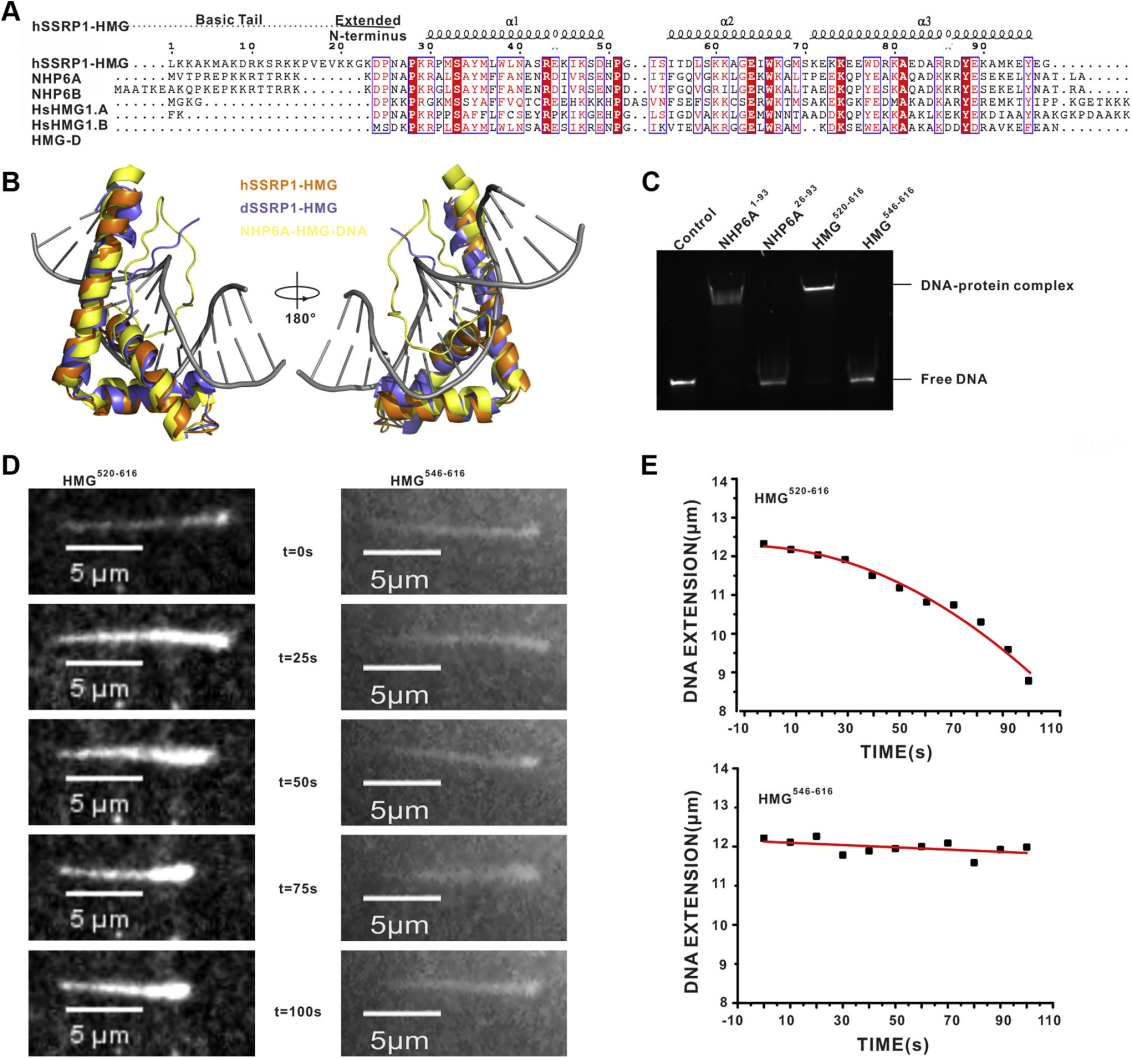
**DNA binding assays of hSSRP1-HMG domain.***A*, sequence alignment of hSSRP1-HMG with HMG1/2 subfamily members. The alignment was created by PROCHECK and visualized by ESPript. The basic tail and extended N-terminus are labeled. *B*, the structure of hSSRP1-HMG superimposing with the structures of NHP6A-HMG-DNA complex and dSSRP1-HMG. *C*, the analysis of N terminus of hSSRP1-HMG in DNA binding by EMSA. *D*, single-molecule visualization of λ DNA compaction induced by hSSRP1-HMG^520–616^ and hSSRP1-HMG^546–616^. The frames at the indicated time points of a representative single DNA molecule from the video recorded at each protein are plotted as a montage. *E*, the compaction of a single DNA molecule by hSSRP1-HMG^520–616^ and hSSRP1-HMG^546–616^. The data are given by *black points*, and the exponential fit is represented by a *red line*. HMG, high-mobility group; hSSRP1, human structure-specific recognition protein.

The C-terminal of hSSRP1 contains a HMG box belonging to the HMG1/2 subfamily, which ubiquitously recognizes DNA nonspecifically (40). The sequence alignment of hSSRP1-HMG with other HMG1/2 subfamily members showed similarity, ranging from 28.6% to 50.0%, with the exception of the N-terminal basic tail in hSSRP1-HMG and NHP6A/B (Fig. 3*A*). We solved the crystal structure of hSSRP1-HMG^546–616^ at 2.0 Å resolution by using molecular replacement. The crystallographic statistics are available in Table 1. The molecular chromatography calibration data showed that hSSRP1-HMG^546–616^ was monomeric in solution (Fig. S5*B*). Similar to the classical HMG structure, hSSRP1-HMG^546–616^ folds into a typical L-shaped fold within three α-helix. Helix α1 (Lys^548^–Asp^568^) and α2 (Ser^573^‒Lys ^586^) wrapped around each other to form the short arm of L, whereas the long arm of L was formed by helix α3 (Lys ^590^–Tyr^614^) (Figs. 3*B* and S5*A*).

Searching a database of protein structures for matches to the hSSRP1-HMG in the Dali server, the solution structures of yeast homolog NHP6A (PDB code: 1CJ7), the NHP6A–DNA complex (PDB code: 1J5N), and the HMG-box domain in SSRP1 from *Drosophila melanogaster* (PDB code: 1WXL) have been reported (42, 52). Comparing with the sequence alignment results, hSSRP1-HMG^546–616^ also shared sequence identity with NHP6A-HMG (38.04%) and SSRP1-HMG from *Drosophila* (dSSRP1-HMG, 43.84%) (Fig. S7). By superimposing these structures with the HMG domain of hSSRP1, we found that the structure of hSSRP1-HMG was highly conserved compared with that of NHP6A-HMG and dSSRP1-HMG, with an RMSD of 1.76 Å on the 60 C^α^ atoms and 2.42 Å on the 54 C^α^ atoms, respectively, with the exception of an additional N-terminal loop in yeast and *Drosophila*. An electrostatic potential comparison between the hSSRP1-HMG^546–616^ domain, NHP6A, and dSSRP1-HMG showed similar charge distributions, indicating that three HMG domains may contain the same DNA binding area (Fig. S5*C*).

Yen *et al.* (53) reported that the N-terminal basic segment of NHP6A is necessary for the efficient binding and bending of DNA. To evaluate the role of the N-terminus of human hSSRP1-HMG in DNA binding, we constructed two hSSRP1-HMG truncations HMG^520–616^ and HMG^546–616^, which were then analyzed by EMSAs and analytical gel filtration. The dsDNA used was rich in GT and labeled with 5-carboxyfluorescein (FAM). The incubation of dsDNA with increasing amounts of hSSRP1-HMG^520–616^ resulted in a progressive reduction in the amount of unbound protein. In contrast, the truncated version of N-terminal loop hSSRP1-HMG^546–616^ showed no interaction with dsDNA (Figs. 3*C* and S5*D*). These results are consistent with the analytical gel-filtration results, wherein a mixture of hSSRP1-HMG^520–616^ with dsDNA showed a complex peak that was eluted earlier than the elution pattern of hSSRP1-HMG^546–616^ (Fig. S5, *E* and *F*). As a positive control, the NHP6A N-terminus deletion was also found to affect dsDNA binding ability (Figs. 3*C* and S5*D*). In summary, the N-terminal tail of hSSRP1-HMG was found to be essential for the interaction with dsDNA.

We also evaluated whether hSSRP1-HMG functions as a DNA chaperone to facilitate the packing of long-chain dsDNA. To this end, single-molecule fluorescence microscopy was performed with no-tagged and GFP-tagged hSSRP1-HMG. The biotin-labeled λ DNA was immobilized by one end on the functionalized surface of a flow cell and extended by a flow at a rate of 50 μl/min. Then, variants of hSSRP1-HMG were pumped into the flow cell to observe their interaction with λ DNA in real time. As shown in the left panel of Figure 3, *D* and *E*, when injecting hSSRP1-HMG^520–616^ into the flow cell, a clear retraction on the length of λ DNA was observed. The decreased length of the λ DNA molecule indicates that hSSRP1-HMG^520–616^ bends DNA. Using GFP-tagged hSSRP1-HMG^520–616^, the GFP fluorescence was observed to be colocalized with λ DNA in a double beam splitting system, suggesting the binding of hSSRP1-HMG^520–616^ to λ DNA (Fig. 3, *D* and *E*, and Video S1). On the contrary, after introducing hSSRP1-HMG^546–616^ in the flow cell, λ DNA did not retract as it did when injecting hSSRP1-HMG^520–616^ (Fig. 3, *D* and *E*, and Video S2). This observation is consistent with the findings in the EMSA experiments. Therefore, these data revealed that the dsDNA-binding ability of hSSRP1 is manipulated by the loop region in the N-terminus of HMG domain.

### CK2 binding sites in hSSRP1

**Figure 4:**
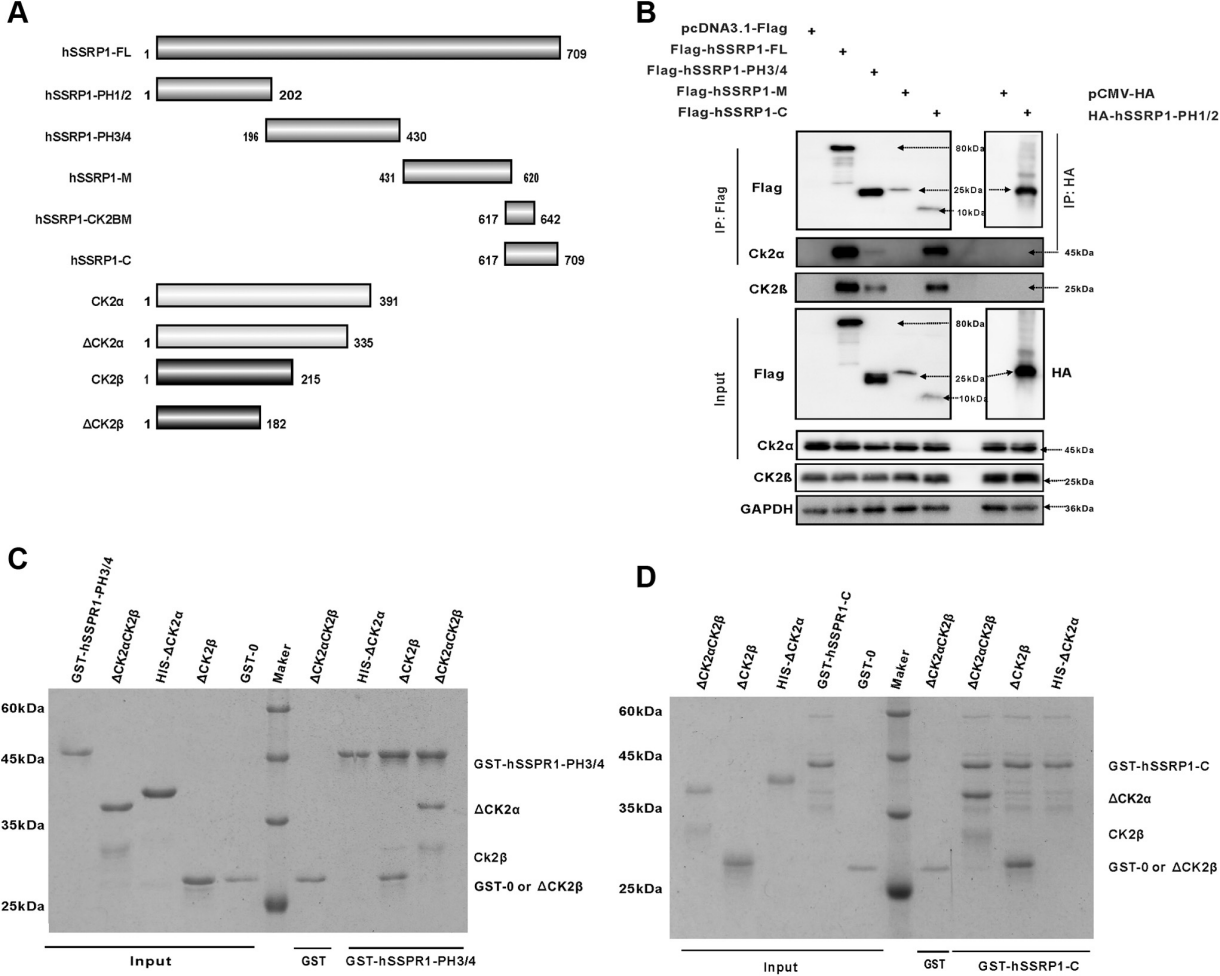
**The binding assays of hSSRP1 with CK2.***A*, schematic of full length and truncation of hSSRP1 and CK2. *B*, coimmunoprecipitation (Co-IP) assays show the interactions between full length and truncation of hSSRP1 with CK2. *C*, pull-down experiment with GST–hSSRP1-PH3/4, △CK2α, △CK2β,and △CK2α/CK2β complex. *D*, pull-down experiment with GST–hSSRP1-C, △CK2β, △CK2α and△CK2α/CK2β complex. The input is the total mixture of beads and proteins, and output is the pull-down. The protein gels are stained by Coomassie blue. CK2, casein kinase II; Fl, full length; hSSRP1, human structure-specific recognition protein 1.

A previous study reported that the regulatory β subunit of CK2 was found to bind specifically to hSSRP1, which is involved in the formation of the hSTP16–SSRP1–CK2 complex (16). To evaluate the specific CK2 binding domain of hSSRP1, flag-hSSRP1 fusion proteins were expressed in cells with either WT hSSRP1 or with four deletion mutants that spanned the length of the protein (Fig. 4*A*). Immunoprecipitation detection indicated that hSSRP1-PH3/4(Gly^196^–Leu^430^) and a disordered region at the C-terminus of hSSRP1 (Gly^617^–Glu^709^ aa) were able to precipitate significant amounts of CK2 (Fig. 4*B*). To identify the CK2 binding region *in vitro*, we performed affinity-tag fusion-based assays. His-tag labeled CK2α and CK2β were prepared with glutathione-S-transferase (GST)-tag labeled hSSRP1-PH3/4 and hSSRP1-C, respectively. The pull-down assay showed that both the hSSRP1-PH3/H4 and C terminal disorder regions were able to interact with CK2β. Furthermore, we identified that the C-terminus of hSSRP1 Gly^617^–Thr^642^ is the key region to bind with CK2 (Fig. S6). Hence, two binding domains of hSSRP1 with CK2 were observed, including hSSRP1-PH3/4 and hSSRP1-C (Gly^617^–Glu^642^) (Figs. 4*C* and S6).

### Location of nuclear localization sequence in hSSRP1

**Figure 5:**
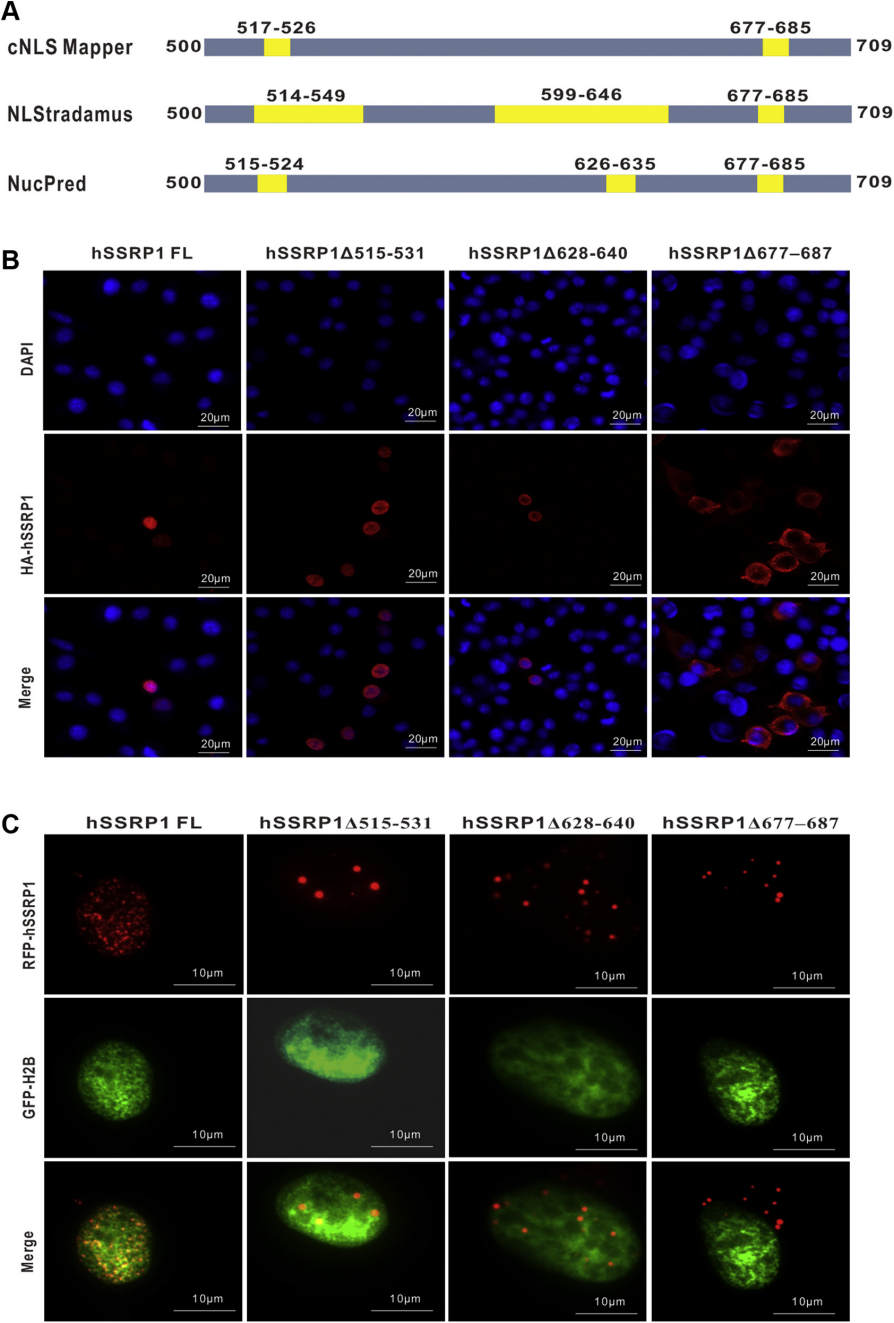
**The prediction and examining of hSSRP1 nuclear localization signal.***A*, the hSSRP1 NLS was predicted by cNLS Mapper, NLStradamus, and Nucpred programs, respectively. The predicted NLS are colored by *yellow*. *B*, the subcellular location of hSSRP1 and its truncated variants. The cells were fixed and immunostained with specific antibodies and Alexa594-conjuagated secondary antibody (*Red*). *C*, the subcellular location of hSSRP1 and its truncated variants with H2B. Transduction of red fluorescent protein (RFP)-tagged hSSRP1full length or variants (*Red*) and GFP-H2B(*Green*) into HepG2 cells were examined by immune fluorescence. DAPI staining was performed to visualize the nuclei (*blue*), and the images were merged. DAPI, 4′, 6-diamidino-2-phenylindole; hSSRP1, human structure-specific recognition protein 1.

hSSRP1 plays an essential role in DNA transcription, replication, and nuclear repair. However, the location of the NLS of hSSRP1 is still unclear. To identify the NLS of hSSRP1 (which is responsible for hSSRP1’s nuclear localization), we analyzed its amino acid sequence using several nuclear localization signal prediction programs. The analysis results from cNLS Mapper (54) showed that the two putative NLS motifs located in Arg^517^‒Ala^526^ and Lys^677^‒Ser^685^ of hSSRP1; Three motifs Glu^514^‒Arg^549^, Lys^599^‒Gly^646^, and Lys^677^–Ser^685^ were predicted as potential NLSs by NLStradamus (55), and Nucpred (56) proposed similar results, including three putative motifs Lys^515^‒Lys^524^, Lys^626–^Val^635^, and Lys^677^–Ser^685^ (Fig. 5*A*).

Based on these predicted results, we then created a series of HA tag and red fluorescent protein (RFP) fusions to determine the role of these sequences, which may have functions with respect to localizing hSSRP1. HepG2 cells were transfected with expression plasmids encoding hSSRP1, hSSRP1Δ515 to 531, hSSRP1Δ628 to 640, and hSSRP1Δ677 to 687 with an HA-epitope tag. Immunofluorescence was performed using 4′, 6-diamidino-2-phenylindole for nuclear staining. As shown in Figure 5*B*, the full-length hSSRP1, hSSRP1Δ515 to 531, and hSSRP1Δ628 to 640 labeled with HA tag were localized to the nucleus of HepG2 cells. The mutant hSSRP1Δ677 to 687 deleting the predicted NLS (Lys^677^‒Asp^687^) abolished its nuclear localization. Furthermore, we performed transient expression of the RFP-hSSRP1 fusion proteins in cells, where GFP-histone H2B accumulated within the nucleus as a control. RFP fusion to either hSSRP1Δ515 to 531 or hSSRP1Δ628 to 640 was found to be fully localized to the nucleus. In contrast, the GFP-hSSRP1Δ677 to 687 fusion protein appeared in the cytoplasm (Fig. 5*C*). Taken together, our data indicate that the putative NLS (Lys^677^‒Asp^687^) exerts high levels of activity in mediating hSSRP1 nuclear transportation.

## Discussion

**Figure 6:**
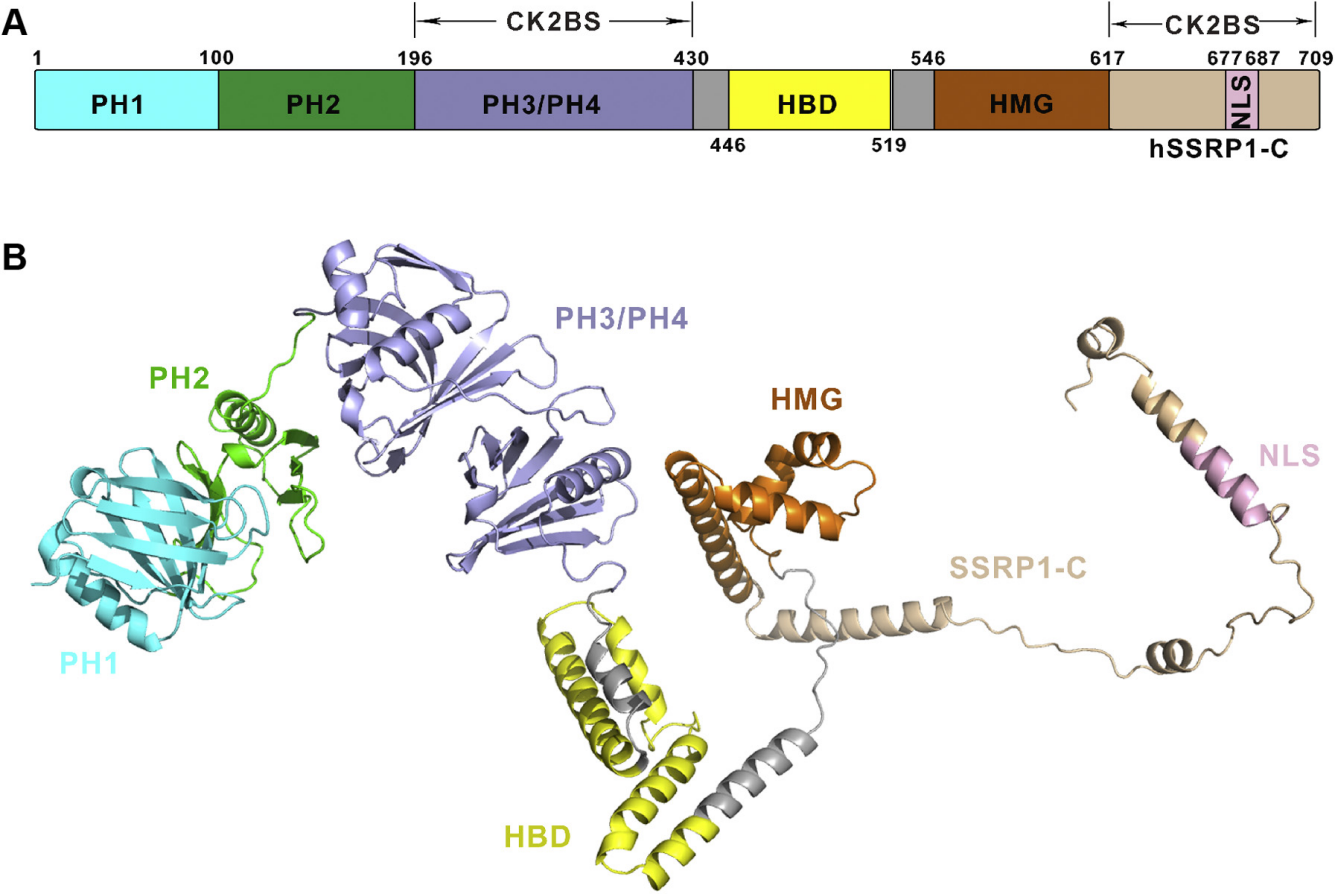
**Overview of hSSRP1 full-length structural model.***A*, a schematic representation of hSSRP1 full-length. PH1 domain, PH2 domain, PH3/4 domain, HBD domain, HMG domain, hSSRP1-C and NLS are shown in *cyan*, *green*, *light blue*, *yellow*, *orange*, *wheat* and *light pink*, respectively. Two CK2 binding sites (CK2BS) were labeled. *B*, a *cartoon* representation of the model of hSSRP1 full-length. CK2, casein kinase II; HBD, H2A–H2B binding region; HMG, high-mobility group; hSSRP1, human structure-specific recognition protein 1; PH, pleckstrin homology.

The present study aimed to determine the structure and functional relationship of full-length hSSRP1, a multiple domain protein that plays diverse roles in transcription, DNA replication, and DNA damage repair. Here, we presented five structural models and three functional motifs covering the full-length hSSRP1, comprising four N-terminal PH domains (PH1–PH4) and an HMG domain. Three specific protein binding motifs of hSSRP1 located in the two intrinsically disordered regions were extensively characterized, including the histone H2A–H2B binding motif, CK2 binding motifs, and an NLS at the end of the C-terminal of hSSRP1(Fig. 6).

The N-terminal tandem PH domain of hSSRP1, composed of four PH domains (PH1–4), is highly conserved in vertebrates, *Drosophila*, *Caenorhabditis elegans*, and yeast, suggesting an early origin and fundamental importance to eukaryotic biology. The previous studies have revealed that PH domains are regularly found in a large variety of proteins with diverse enzymatic or regulatory functions, such as phospholipases, GTPase-regulating proteins, and protein kinases, playing roles in cellular signaling and cytoskeletal organization. In addition, recent structural studies have shown that transcription and DNA repair factors adopt the same folds as Rtt106, p62, and SSRP1 (57). However, hSSRP1 is a unique member of the PH fold superfamily, with four tandem PH domains assembled. Based on our structural models of each PH domain of hSSRP1, the PH1 to 4 subdomains were all found to have a canonical PH fold with a seven-stranded β sandwich closed with a C-terminal α helix. Furthermore, each PH subdomain of hSSRP1 may possess specificities for various binding partners. In a recent study, Falbo *et al.* (58) reported that the N-terminal domain hSSRP1 covering PH1 and PH2 are involved in binding to histone H1. According to our studies, PH1 is unable to interact with histones, including histone H1, indicating that PH2 may contribute to the binding of histone H1. In humans, the histone H1 family includes 11 different H1 variants with seven somatic subtypes (59). The mechanism of histone H1 binding with hSSRP1 is still unclear; further assays are required to uncover the detailed mechanisms. The second PH2 subdomain is required for hSSRP1 interaction with Spt16, forming the FACT complex. Without Spt16, the single PH2 subdomain is unstable and aggregates at high concentrations *in vitro*. The truncated hSSRP1 without the PH2 subdomain completely lost its binding ability with Spt16 (60). These results are also consistent with the crystal structure of the Pob3 Pob3-N/Spt16 complex, which revealed that Pob3-PH2, but not Pob3-PH1, is responsible for heterodimerization with the dimerization domain of Spt16 (37). The subdomains of PH3 and PH4 have been reported to have a tandem PH domain, which prefers to bind dsDNA instead of histones (39). Our results found that hSSRP1-PH3/4 could interact with multiple binding partners, including dsDNA and CK2. Interestingly, this domain only binds 30 bp dsDNA but not 15 bp dsDNA, and not even long dsDNA fragments could bind with hSSRP1-PH3/4. These results may explain why hSSRP1-PH3/4 is involved in limited DNA binding with nucleosomes (38). Recently, Liu *et al.* reported two cryo-EM structures of human FACT in complex with partially assembled nucleosomes, in which four PH domains of hSSRP1 gathered together, forming multiple interfaces with the DNA in the nucleosome core. Therefore, the N-fragment of hSSRP1 (1–430) was designated as a tandem PH domain, which is composed of an N-terminal domain (PH1), Spt16-binding domain (PH2), and middle domain (PH3–4).

Following the N-terminal tandem PH domain, a histone H2A–H2B binding motif is located within the unstructured acidic region. A previous study found that Pob3, the hSSRP1 yeast homolog, contains an H2A–H2B binding region in its C-terminus (50). Based on the sequence alignment results, we found that the H2A–H2B binding motif in hSSRP1 is characterized by a unique sequence with (D/E) XXФ, where Ф is Phe or Tyr, and X is any residue, which is not only highly conserved in hSSRP1 homologs, but is also found in the histone chaperones ANP32E and SWR1. These results indicate that these histone chaperone proteins interact with H2A–H2B by using a (D/E) XXФ motif within an intrinsically disordered region to engage the H2A–H2B dimer. A model of the histone binding motif of hSSRP1 in complex with an H2A–H2B dimer showed that the acidic residue Asp^479^ and the hydrophobic residue Phe^482^ serve as the helix capping and aromatic anchor residues, respectively. The mutations in the hSSRP1 (D/E) XXФ motif inhibited histone H2A–H2B binding with hSSRP1 *in vitro*, supporting a model in which hSSRP1 acts as a histone H2A–H2B chaperone protein to promote H2A–H2B dimer eviction.

The HMG domain of hSSRP1, the last folded domain after the H2A–H2B binding site of hSSRP1, is a highly conserved domain that mediates the DNA binding of many proteins. Previous studies have divided the HMG domain into two subfamilies based on the differences in amino-acid sequence and specificity of DNA binding. The first class generally comprises transcription factors that bind to DNA with sequence specificity. On the other hand, the second class of HMG box proteins is more abundant, containing two or more tandem HMG boxes and binds DNA with little or no sequence specificity. Therefore, the HMG domain of hSSRP1 is similar to HMG proteins in class two but contains only one HMG box. Similar to other nonsequence-specific HMG proteins, the HMG-hSSRP1 domain folds into an L-shaped structure *via* three helices. A highly basic region that precedes the HMG domain of hSSRP1 is essential for DNA binding and the formation of the HMG–DNA complex. Indeed, the removal of the N-terminal tail from hSSRP1-HMG completely abolished the dsDNA-binding ability, indicating that the N-terminal ends were beyond the minimal fold, which is necessary for high-affinity binding to DNA.

The functional role of the C-terminal disordered region (CTD) after the HMG domain in hSSRP1 has been previously found to be important for cell proliferation and histone binding (61). In this study, we report two biological roles associated with the CTD, including one of two CK2 binding sites and a nuclear localization sequence. In addition to the hSSRP1-PH3/4 domain located in the N-terminal tandem PH domain binding with CK2, the second CK2 binding site located in the CTD region of hSSRP1 was observed because Keller *et al.* (16) first reported that CK2 binds to the hSSRP1. Another feature is that CTD contains an NLS to mediate the nuclear entry of hSSRP1. This result ruled out the possibility that the NLS of hSSRP1 was located within the basic N-terminal region of the HMG domain (30). Considering previous studies by Rottgers *et al.* (62) and Hoffmann *et al.* (51), who reported that the basic region adjacent to the HMG domain of hSSRP1 from maize was sufficient for nuclear targeting and the NLS in yeast Pob3 is located at the C-terminus (544‒552), we propose that the location of the NLS in hSSRP1 is not highly conserved in SSRP1 homologs because of the differences in species.

From a structural point of view, the results presented here are expected to combine all of the puzzle pieces to show the entire structure of hSSRP1. The N- to C-terminal order of domains and motifs in hSSRP1 are connected through the three-dimensional structures and their functional relationships. In particular, we expect to answer the question of how hSSRP1 uses multiple domains to fulfill its diverse role in transcription initiation, DNA replication, and DNA repair. However, the data obtained here are still not sufficient to answer this question. Further work is required to characterize the nature and multiple functional roles of hSSRP1 and its associated complexes.

## Experimental procedures

### Plasmid construction, protein expression, and purification

cDNA fragments encoding hSSRP1-PH1 (Met^1^–Asp^100^), -PH2 (Leu^101^–Thr^195^), and -PH3/4 (Gly^196^–Asn^430^), -PH1-4(Met^1^–Asn^430^), hSSRP1-HBD (442–511aa), hSSRP1-HMGD (Leu^520^–Gly^616^ and Ala^546^– Gly^616^), and hSSRP1-C (Gly^617^–Glu^709^) were amplified from the full-length hSSRP1 cDNA (OriGene) by PCR. Each of the cDNAs was cloned into the pGEX6p-1 and pET28a-sumo vectors. The yeast NHP6A cDNA was extracted from the yeast genome by RT-PCR and cloned into the PET28a vector. Human H2A and H2B gene mutations were generated using a QuikChange Site-directed Mutagenesis kit (Stratagene) according to the manufacturer's protocol. For expression by transient transfection, the full-length hSSRP1 and putative hSSRP1-NLS truncations (hSSRP1Δ515–531, hSSRP1Δ628–640, and hSSRP1Δ677–687) were separately cloned into PCMV-HA and pDsRED-N1 vectors to express HA- or RFP-tagged proteins. The histone H2B gene was cloned into the pDsEGFP-N1 vector to express GFP-tagged protein. The recombinant plasmids were confirmed by DNA sequencing (Invitrogen) and transformed into *Escherichia coli* Rosetta (DE3) (Transgene); then, the recombinant proteins were purified by GST affinity resin (GE Healthcare) or nickel-nitrilotriacetic acid (Qiagen) affinity chromatography. The GST was cleaved with PreScission Protease (GE Healthcare). The cleaved proteins were further purified by anion exchange and size-exclusion chromatography. Human CK2α and CK2β genes were cloned into a pETDuet-1 vector, respectively. Human H2A-H2B complex, CK2α/CK2β, and H1.1 were purified, as described (63, 64, 65).

### Crystallization and data collection

The protein samples were concentrated to 20 to 40 mg/ml for crystallization trials using the hanging drop vapor diffusion method by mixing 1 μl protein solution and 1 μl reservoir solution at 287K. The hSSRP1-PH1 crystals with suitable X-ray diffraction were grown in a reservoir solution that contained 30% w/v polyethylene glycol 1500 and 40% 1,2-butanediol (Hampton Research). The hSSRP1-PH3/4 protein crystallized with 10 mm MES pH 4.0, 12.5% 2-propanol, 20% PEG2000, and sodium iodide as additive reagents. To solve the phase problem of hSSRP1-PH1 and hSSRP1-PH3/4, selenomethionine-labeled proteins were prepared by a classical protocol (66), which crystallized under similar conditions. The selenomethionine single-wavelength anomalous dispersion data set of hSSRP1-PH1 and hSSRP1-PH3/4 at the selenium peak wavelength was collected at the Shanghai Synchrotron Radiation Facility beamline BL17U1. hSSRP1-HMGD protein crystals were grown under the precipitant condition with 2.2 M DL-malic acid pH7.0, 0.1 M BIS-TRIS propane pH7.0. hSSRP1-HMGD diffraction data were collected on a home X-ray resource Oxford diffraction KM4 Xcalibur 2 and processed using CrysAlisPro.

### Structure determination and refinement

The diffraction data set was processed and scaled by using the HKL2000 package. The selenium atoms were located and refined, and the single-wavelength anomalous dispersion data phases were calculated and substantially improved by solvent flattening using the PHENIX program. The hSSRP1-HMGD structure was solved using molecular replacement by Phaser in the CCP4 program suite with the crystal structure of a member of the HMG family (PDB code: 3FGH) as an initial search model. The cycles of refinement and model building were carried out using the REFMAC5 (67), Phenix (68), and COOT (69) software programs. The model geometry was verified using the program MolProbity (70). Structural figures were drawn using the program PyMOL (DeLano Scientific). The data collection and refinement statistics of hSSRP1-PH1, hSSRP1-PH3/4, and hSSRP1-HMGD are shown in Table 1.

### Electrophoretic mobility shift assays

EMSAs were performed, as described previously, (71) to detect the DNA-binding ability of hSSRP1-HMGD^520–616^, HMGD^546–616^, NHP6A^1–93^, and NHP6A^26–93^. The oligonucleotide sequence used was 5′FAM-GGGGTGATTGTTCAG-3′ (Sangon). The labeled double-stranded DNA was annealed at 98 °C and gradually cooled to room temperature. Oligonucleotides (5 μM) were incubated for 30 min on ice with purified hSSRP1-HMGD in binding buffer (137 mM NaCl, 2.7 mM KCl, 10 mM Na_2_HPO_4_, and 2 mM KH_2_PO_4_ pH 7.4). The complex was separated on a 12% native polyacrylamide gel (25 mM Tris, 192 mM glycine, pH 8.25). The bands were detected with Quantum ST5 (Brand).

### Isothermal titration calorimetry

ITC experiments were performed at 16 °C using the MicroCal ITC200 system (GE healthcare). For ITC, the final purification step was gel filtration chromatography in 20 mM Tris (pH 8.0) and 200 mM NaCl. The titrations included an initial injection volume of 0.4 μl (omitted from analysis) and 20 injections of 2 μl spaced at intervals of 180 s. The data were analyzed using Origin 7 software. All the reactions were performed at least in triplicate.

### Single-molecule fluorescence imaging

To obtain information about the binding kinetics of hSSRP1-HMGD to DNA, biotin-labeled lambda-phage DNA molecules attached to beads were held by an optical trap and extended by flowing the buffer in a two-channel flow cell, as previously described (72, 73). The binding buffer used in these experiments contained 10 mM Tris, 20 mM EDTA, 25 mM KCl, 0.2 mg/l bovine serum albumin, and 10 mM MgCl_2_. λ DNA was stained with SYTOX dye, allowing the compaction to be observed using fluorescence microscopy. The molecule was then moved back to the DNA side of the flow cell (which was protein free), and the decompaction of the molecule was observed as protein left in it, ultimately returning to its original length.

### Pull-down experiments

The mixture contained 40 μg of GST or GST-tagged protein and 120 μg of bait proteins. The proteins were mixed with glutathione Sepharose 4B resin in 1 ml of pull-down buffer (PB: 20 mM Tris, pH 8.0, 150 mM NaCl, and 0.005% Triton X-100) and incubated for 30 min on a rotator. After extensive washing with PB buffer, the bound proteins were separated by SDS/PAGE and visualized by Coomassie staining. Each experiment was repeated at least once and checked for consistency.

### Cell culture and immunoprecipitation

Various hSSRP1 expression plasmids were severally transfected by PEI into cultured HeLa cells. 48 h later, the cells were harvested in PBS by a cell scraper and centrifuged at 4 °C using 300*g* for 10 min. The pellets were resuspended in 1 ml extraction buffer (10 mM Hepes pH 7.9, 10 mM KCl, 1.5 mM MgCl2, 0.34 M sucrose, and 10% Glycerin, with 0.2% NP40; protease inhibitors and phosphatase inhibitors were added before use). The samples were incubated for 20 min on ice while occasionally rotating, and they were then centrifuged at 4 °C, 6500*g* for 5 min. The supernatant (the cytoplasm) was collected completely and carefully with a 1 ml pipette. Then, 100 μl of the supernatant was separated as input, and the remaining supernatant was incubated overnight at 4 °C either with 35 μl of Flag/GFP/HA conjugated magnetic beads prewashed with coimmunoprecipitation buffer three times or with 1 μg of relevant primary antibodies (CK2α). After overnight incubation, either the protein-magnetic beads complex was washed five times with IP washing buffer, or the protein-antibody complex was incubated with 35 μl of magnetic beads conjugated with prewashed protein A/G at 4 °C for 3 h. The precipitated proteins were dissolved in 1× SDS sample buffer, boiled at 95 °C for 5 min, and subjected to Western blot analysis. The IP samples were resolved on a 12.5% SDS/PAGE gel, and the proteins were transferred to PVDF membranes for Western blotting. The proteins were detected using specific antibodies. The antibodies are listed below: Flag-tag antibody (Huabio#M1403-2), GFP-Tag antibody (Proteintech#50430-2-AP), HA-Tag antibody (Proteintech#51064-2-AP), hSSRP1 antibody (Biolegend#609701), hSSRP1 antibody (Abcam#ab137034), CSNK2A1(CK2α) antibody (Proteintech#10992-1-AP), and CSNK2B(CK2β) antibody (Abclonal#A2869).

### Immunofluorescence staining

The antibodies used for immunofluorescence are listed below: anti-HA tag antibody (AbCam ab9110) and Goat anti-rabbit lgG (AbCam ab205718). The HepG2 cells (epithelial hepatocellular carcinoma, ATCC) were routinely maintained with regular DMEM supplemented with 10% fetal bovine serum (Invitrogen). Transient transfection was carried out using Lipofectamine 2000 (Invitrogen) according to the instructions of the manufacturer. 48 h later, the cells were fixed in 4% paraformaldehyde for 20 min at 4 °C followed by permeabilization with 0.5% Triton-X 100 at 4 °C for 20 min. The cells were blocked in 5% nonfat dry milk for 30 min at 4 °C and subsequently probed using HA-tagged antibody raised against recombinant hSSRP1 (Santa Cruz Biotechnologies) at a 1:100 dilution at 4 °C overnight. After being washed three times with PBS, it was probed with goat anti-rabbit IgG (H + L) human serum adsorbed antibody conjugated to Alexa594 (Life Technologies) at a 1:200 dilution for 30 min at 37 °C. The cells were washed three times with PBS after being incubated with 0.5 μg/ml 4′, 6-diamidino-2-phenylindole for 10 min. The images were acquired using a Leica SP5 system.

## Data availability

The atomic coordinates and structure factors of hSSRP1-PH1, -PH3/4, and HMG were submitted to RCSB Protein Data Bank, with accession codes 6L1R, 6L1E, and 6L34, respectively.

## Supporting information

This article contains supporting information.

## Conflict of interest

The authors declare that they have no conflicts of interest with the contents of this article.
