# Supplementary material for: Structural insights into multifunctionality of human FACT complex subunit hSSRP1
Source: J Biol Chem. 2021 Oct 28;297(6):101360. doi: 10.1016/j.jbc.2021.101360 (PMC8639466; doi:10.1016/j.jbc.2021.101360)

# Supplementary information

# Structural insights into multifunctionality of human FACT complex subunit hSSRP1

Xuehui Li^a#^, Huiyan Li^a#^, Qian Jing^b#^, Mengxue Wang^c^, Tingting Hu^d^, Li Li^a^, Qiuping Zhang^a^, Mengxin Liu^a^, Yu Vincent Fu^c^, Junhong Han^b*^, Dan Su^a,e*^

**Supplementary table**

Table S1. Structure comparison details of hSSRP1-PH1 and hSSRP1-PH3/4

|  | hSSRP1-PH1 | hSSRP1-PH3/4 | RMSD |
| --- | --- | --- | --- |
| 4KHB.D.1aa-97aa | + | - | 1.443 |
| 3F5R | + | - | 1.846 |
| 4IFS | - | + | 0.787 |
| 4PQ0 | - | + | 1.201 |
| 3GYP | - | + | 2.60 |

The calculation of the rmsd value is based on all C^a^ atoms

**Supplementary figures and legends**

Figure S1. The properties of hSSRP1-PH1. **(A)** The structural superposition of hSSRP1-PH1(Orange), yeast Pob3-N (cyan, PDB ID: 3F5R), Chaetomium thermophilum Pob3-N (paleyellow, PDB ID: 4KHB.D). **(B)** The SEC assay is used to evaluate the molecular weight of hSSRP1-PH1. The black line is from commercial molecular weight standards, and the hSSRP1-PH1 chromatogram is colored red.**(C)**SEC analysis of the interaction between hSSRP1-PH1 and H2A–H2B.**(D)** SEC analysis of the interaction between hSSRP1-PH1 and H3H4. **(E)** GST pull down of GST-SSRP1-PH1 with immobilized H1.1 **(F)** IP assay of SSRP1-PH1 and Flag-H1.1 **(G)** EMSA results of hSSRP1-PH1 with 30bp linker dsDNA. In each lane, dsDNA was incubated with increasing amounts of hSSRP1-PH1 (1:1, 1:10, 1:20, 1:50, 1:100, 1:200, respectively).


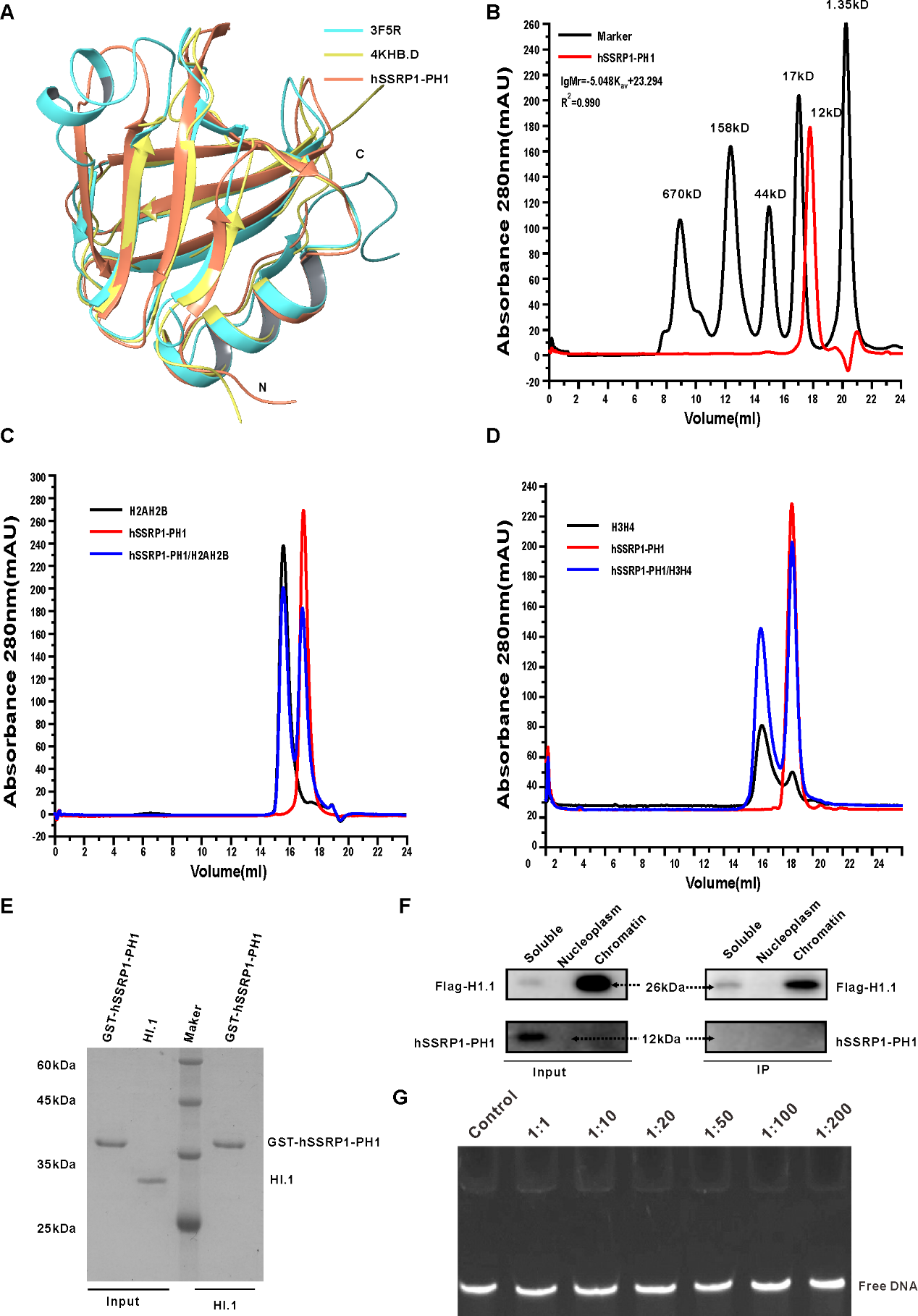


Figure S2. The properties of hSSRP1-PH2. **(A)** The SEC assay is used to evaluate the molecular weight of hSSRP1-PH2. The black line is from commercial molecular weight standards, and the hSSRP1-PH2 chromatogram is colored red. **(B)** The Ramachandran plot of hSSRP1-PH2. **(C)** Assessment of quality of hSSPR1-PH2 model by QMEAN4 scoring function. Predicted QMEAN4 and Z-scores, shown with red star, of hSSPR1-PH2 model were within the limits of good quality structures. **(D)** ProSa Z-score (−3.05) plot of the hSSRP1-PH2. **(E)** EMSA results of hSSRP1-PH2 with 15bp ssDNA,30bp ssDNA, 15bp dsDNA and 30bp dsDNA. **(F)** SEC analysis of the interaction between hSSRP1-PH1, hSSRP1-PH2 and hSSRP1-PH3/4.


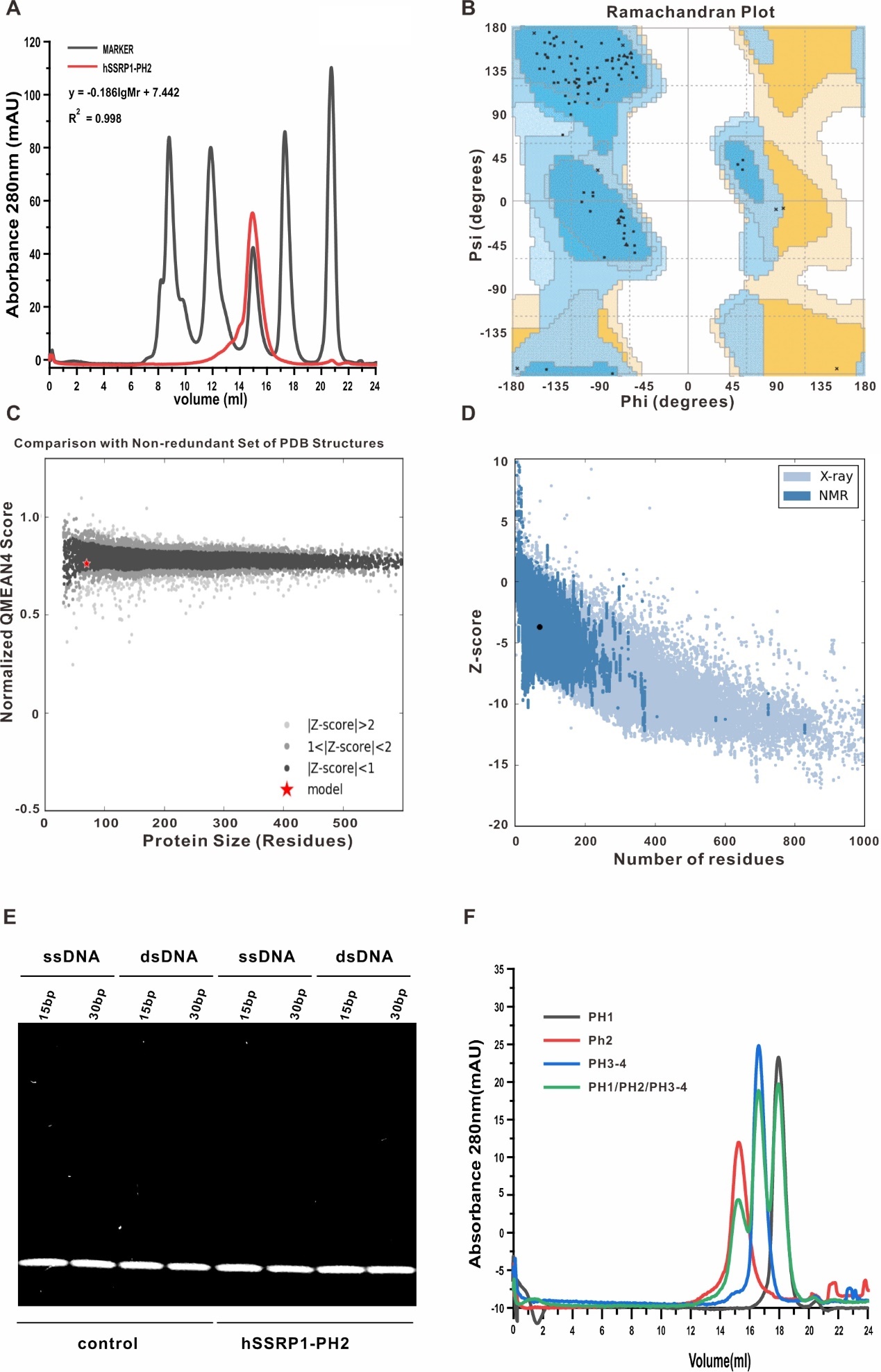


Figure S3. The properties of hSSRP1-PH3/4. **(A)** The structural superposition of hSSRP1-PH3/4(paleyellow), reported human SSRP1-PH3/4 (slyblue, PDB ID: 4IFS), its yeast homologue Pob3-MD (limegreen, PDB ID: 4PQ0), the related protein Rtt106-MD (gray90, PDB ID: 3GYP). **(B)** The SEC assay is used to evaluate the molecular weight of hSSRP1-PH3/4. The black line is from commercial molecular weight standards, and the hSSRP1-PH3/4 chromatogram is colored red. **(C)** SEC analysis of the interaction between hSSRP1-PH3/4 and H2A–H2B. **(D)**SEC analysis of the interaction between hSSRP1-PH3/4 and H3-H4. **(E)** EMSA results of hSSRP1-PH3/4 with 15bp linker dsDNA. In each lane, dsDNA was incubated with increasing amounts of hSSRP1-PH3/4 (1:1, 1:10, 1:20, 1:50, 1:100, 1:200, respectively). **(F)** EMSA results of hSSRP1-PH3/4 with 30bp linker dsDNA. In each lane, dsDNA was incubated with increasing amounts of hSSRP1-PH3/4 (1:1, 1:10, 1:20, 1:50, 1:100, 1:200, respectively). **(G)** Sequential photographs of DNA digestion at 0, 25, 50, 75 and 100s.


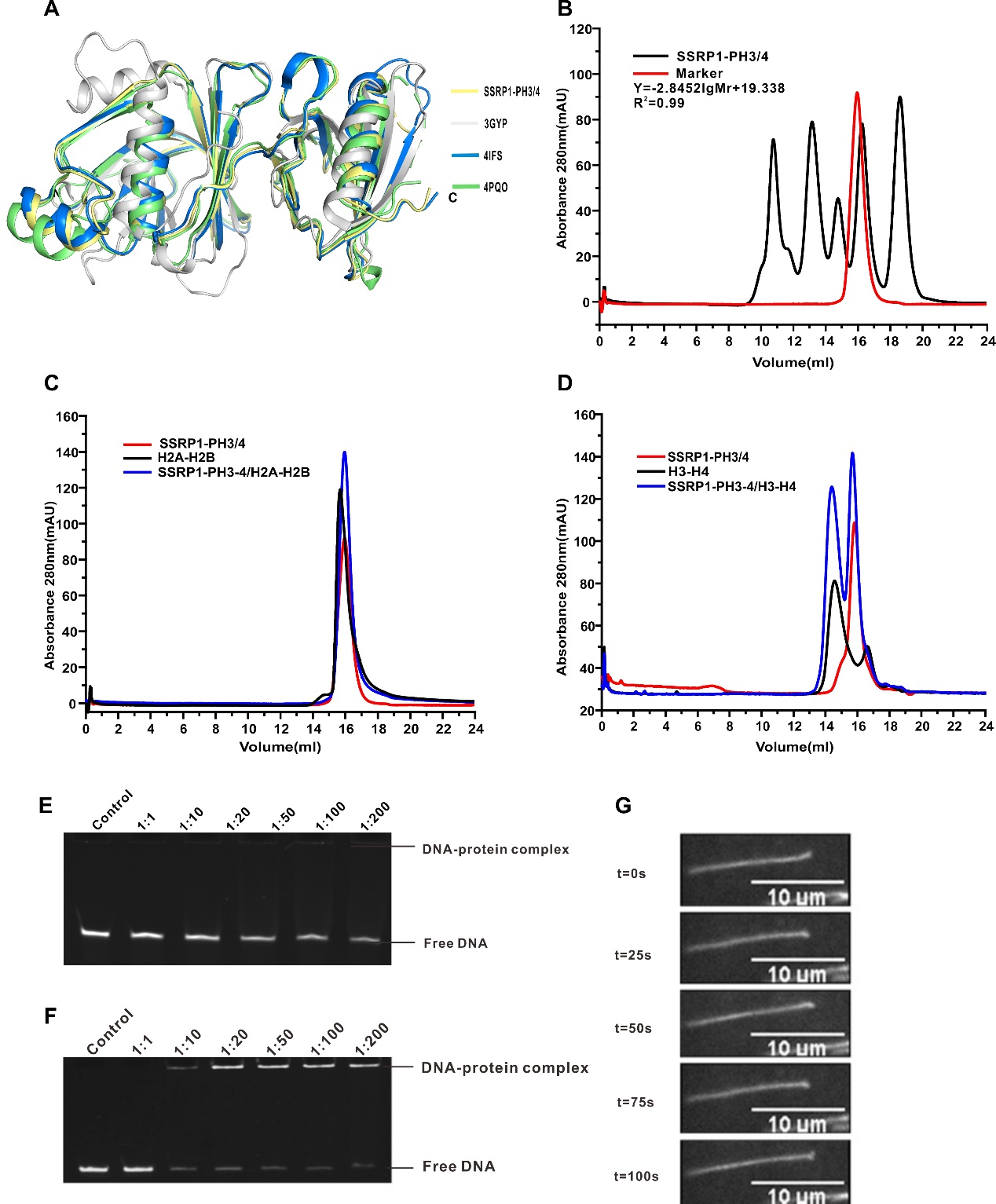


Figure S4. **(A)** SEC analysis of the interaction between hSSRP1-HBD and H2A–H2B.**(B)** SEC analysis of the interaction between hSSRP1-HBD and H2A–H2B^27-126^. **(C-G)** mutant hSSRP1^476-483^ titrated into mutants H2A–H2B-R80E, H2A–H2B-I54A, H2A–H2B-Y43A, H2A–H2B-M59A and H2A–H2B-Y43A /M59A respectively.


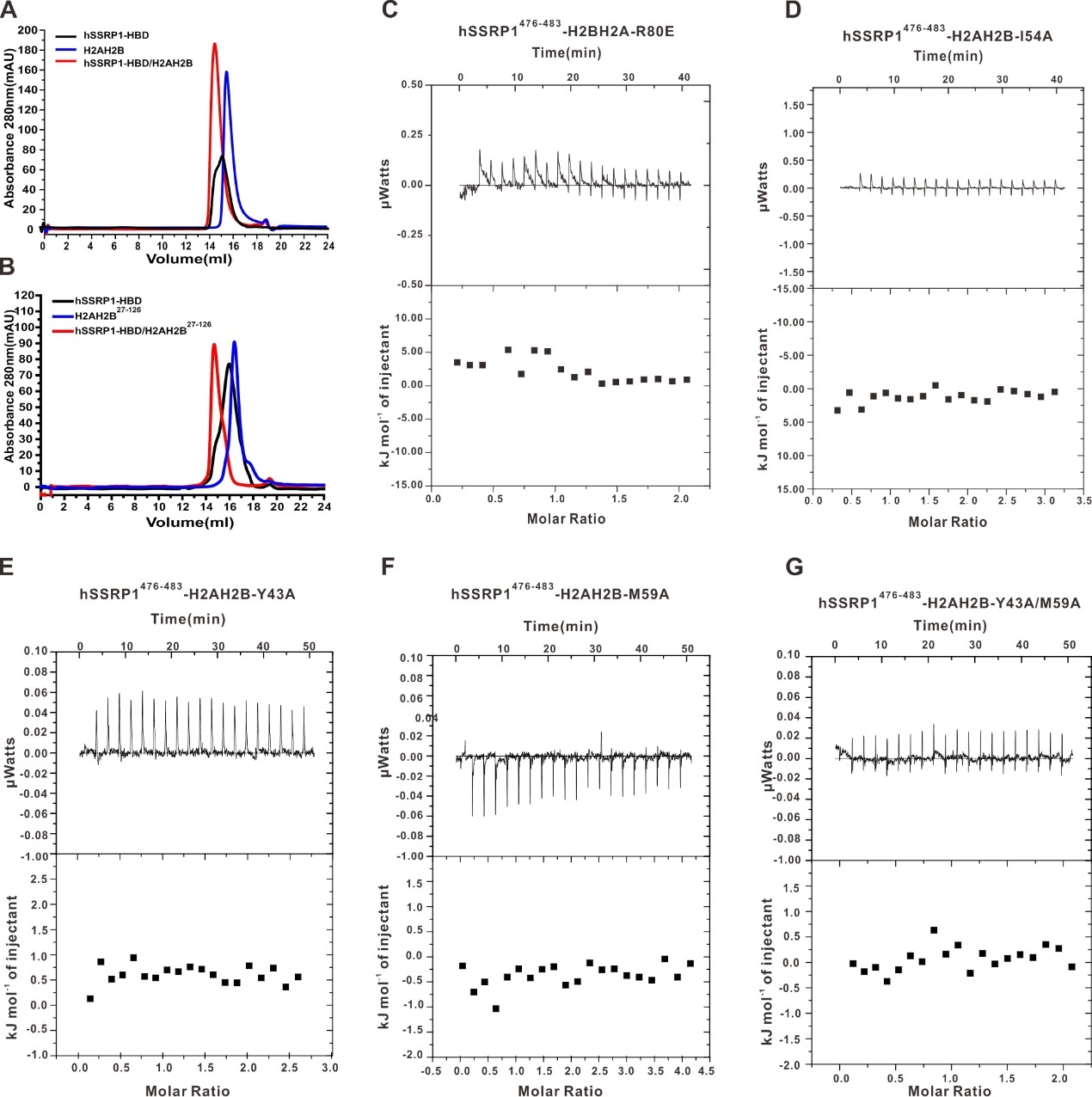


Figure S5. The properties of hSSRP1-HMG. **(A)** The crystal structure of hSSRP1-HMG. The secondary structural elements are labelled. **(B)** The SEC assay is used to evaluate the molecular weight of hSSRP1-HMG. The black line is from commercial molecular weight standards, and the hSSRP1-HMG chromatogram is colored red. **(C)**The electronic potential surface of hSSRP1-HMG, NHP6A-HMG and dSSRP1-HMG. **(D)** EMSA results of hSSRP1-HMG^520-616^, hSSRP1-HMG^546-616^, NHP6A^1-93^ and NHP6A-^26-93^ with 15bp linker dsDNA. In each lane, dsDNA was incubated with increasing amounts of proteins (1:1, 1:5, 1:10, 1:20, 1:50, respectively). **(E)** SEC analysis of the interaction between hSSRP1-HMG^520-616^ and dsDNA. **(F)** SEC analysis of the interaction between hSSRP1-HMG^546-616^ and dsDNA.


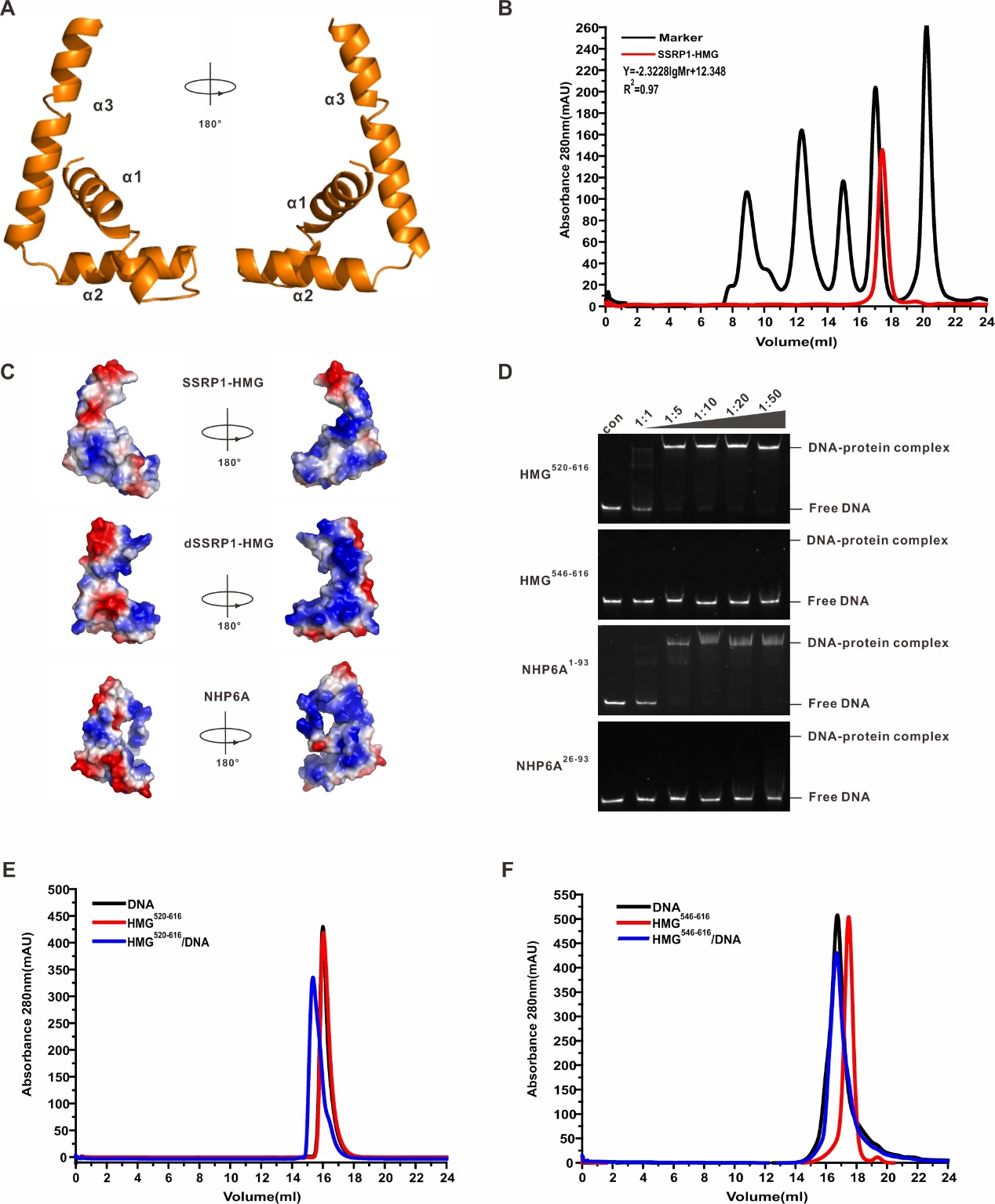


Figure S6. Pull-down experiment with GST–hSSRP1^617-642^ and △CK2β.


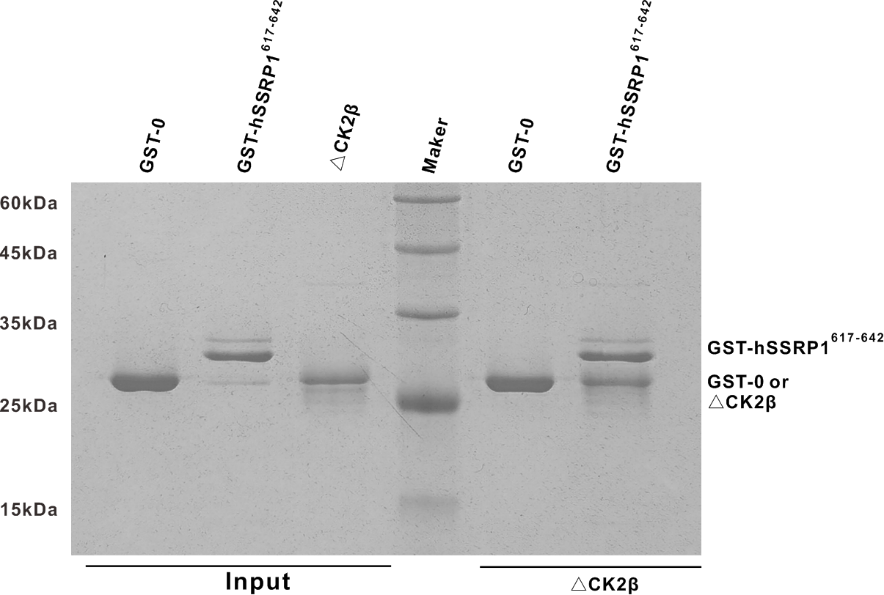


Figure S7. Sequence alignment of hSSRP1 with its homologues.


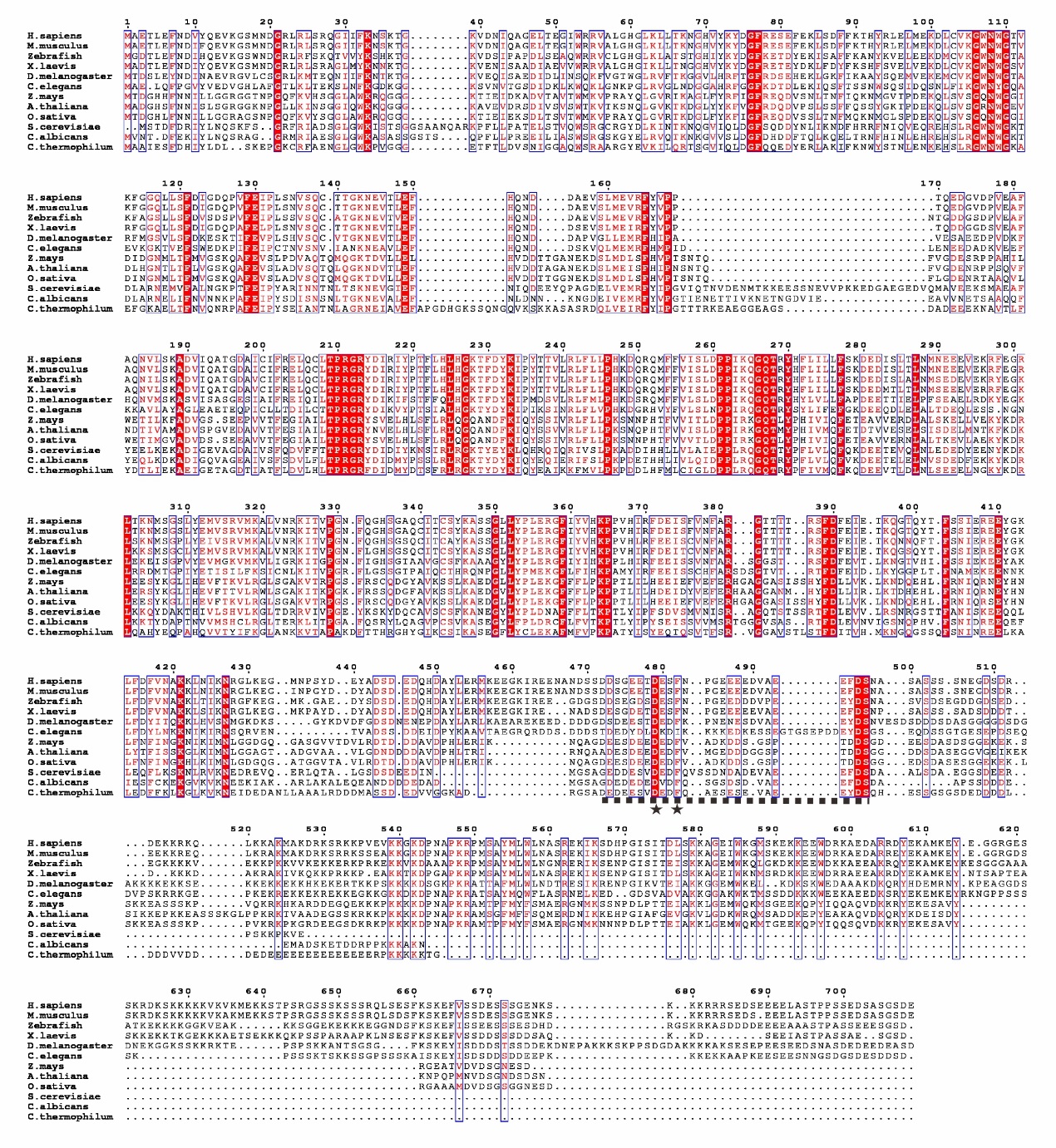

Supplement: Supplemental Figures S1–S7 and Table S1 [file mmc3.docx]
